# When COVID-19 Met Families Living in Armed-Conflict Zones: The Importance of Maternal Trauma and Child Self-Regulation

**DOI:** 10.3389/fpsyt.2022.718455

**Published:** 2022-03-14

**Authors:** Kinneret Levavi, Porat Yakov, Alison Pike, Kirby Deater-Deckard, Amnon Hadar, Guy Bar, Miron Froimovici, Naama Atzaba-Poria

**Affiliations:** ^1^Duet Center, Department of Psychology, Ben-Gurion University of the Negev, Be'er Sheva, Israel; ^2^School of Psychology, University of Sussex, Brighton, United Kingdom; ^3^Department of Psychological and Brain Sciences, University of Massachusetts Amherst, Amherst, MA, United States; ^4^Clalit Health Services, Be'er Sheva, Israel; ^5^Fertility and IVF Unit, Faculty of Health Sciences, Soroka University Medical Center, Ben-Gurion University of the Negev, Be'er Sheva, Israel

**Keywords:** armed conflict zone, adverse effects, COVID-19, maternal PTSS, child self-regulation

## Abstract

The COVID-19 outbreak began in Israel at the end of February 2020, and on March 17, 2020, a general lockdown was announced. Families were instructed to stay at home and schools and non-essential businesses were closed. Aiming to understand how families who were already living in areas of high exposure to armed conflict would be affected by another external stressful condition, data were collected before and after the outbreak. Mothers and children (aged 10–45 months) were recruited from areas with high (*n* = 40) and low (*n* = 78) exposure to armed conflict. Mothers reported on their posttraumatic stress symptoms (PTSS) and on their child's effortful control tendencies prior to the outbreak. Toward the end of the first lockdown, mothers were interviewed regarding adverse effects of the outbreak on their family. No group differences were found for maternal perceptions of adverse effects of COVID-19. However, a moderation model was revealed, indicating that maternal PTSS as well as child effortful control predicted adverse effects of COVID-19 only in the high-exposure group. Results are discussed considering cumulative stress and risk factors.

## Introduction

Exposure of families to armed conflict and political violence is a worldwide problem, currently affecting more than one in 10 children globally (1). Families living in areas of armed conflict experience ongoing exposure to attacks that target civilian areas, creating an uncertain, and often chaotic, reality, which increases vulnerability to mental health difficulties for both parents and children (2). Studies from around the world have concluded that exposure to any type of armed conflict has severe consequences for children at any age, ranging from difficulties in socioemotional development to psychopathological disorders (3). Children are at risk for maladjustment, not only while the violence is occurring, but for years afterward (4, 5). Furthermore, chronic exposure to armed conflict compared to acute episodes was found to have more negative implications for child emotional development and behavioral problems (3, 5–7). A possible explanation is that with chronic exposure, children and parents live in unpredictable and undefined situations for longer periods, which may elicit stress and deplete their internal mental resources (2).

Researchers suggest that during early childhood, trauma due to exposure to armed conflict can be manifested in difficulties in all developmental domains (7–9). Furthermore, because young children's emotional and mental states during their first years of life have a major impact on their normative developmental processes, exposure to external chronic stressors, such as armed conflict, may have a long-lasting effect on future developmental achievements (10, 11).

Interestingly, cumulative evidence shows that young children are affected not only by direct exposure to armed conflict, but more significantly indirectly by the effect of exposure on their primary caregivers (7, 12, 13). Slone and Mann (7) showed that among various studies conducted in war or armed-conflict zones, there was a strong link between child adjustment and parental functioning and mental state. Moreover, it was shown that child maladjustment was more dependent on parental factors, such as maternal mental health, than on the severity of exposure itself (14, 15).

Parenting in an armed-conflict zone is a challenging task; research indicates that parents who are exposed to armed conflict are highly vulnerable to psychopathology, such as post-traumatic stress disorder (PTSD), depression, and anxiety (16–18). Because parents experience chronic uncertainty they must repeatedly adapt to an unexpected reality, which puts them in a constant state of vigilance and dilutes their internal resources (2). Researchers suggest that the loss of mental resources may have long-term consequences and result in helpless feelings, which affect the parents' capacity to offer trustful states of mind to their child. Moreover, it impairs parental emotional regulation, which, in turn, affects parenting practices (2, 19), thus affecting the family climate and resulting in additional stress for the family system (20).

In this study, we aimed to examine how families who are already living in a chronic state of exposure to armed conflict are affected by a new external threat, namely the coronavirus disease-19 (COVID-19) pandemic. We examined families living in the Gaza vicinity who were exposed to continuous armed conflict. The Gaza vicinity is an area in the southern part of Israel, several kilometers from the border of the Gaza strip. Due to political issues, the population living in this area has (for decades) experienced ongoing missile attacks, military activity within civilian surroundings, and the existence of cross-border tunnels used for terror attacks in proximity to their homes. Since the time of data collection (2018–2020), over 2,000 rockets have been launched from Gaza to the Gaza vicinity (21). To warn and protect the residents, an alarm is sounded whenever a rocket is launched, giving residents only a few seconds to find shelter, which is particularly challenging for parents of toddlers who are more dependent on their parents, physically and mentally, as opposed to older children who are more independent and self-regulated (22).

This chronic exposure carries a significant psychological cost for both parents and children (3). It is unknown, however, whether living in stressful conditions and learning to follow specific rules (which may often limit the mobility of the family) is a protective factor or, alternatively, a risk factor when having to manage additional stress. Taking advantage of the unique situation that the COVID-19 pandemic created in Israel, our study aims to explore what happens when new external stress is added to the stress of armed conflict. Will these families have adequate resources to deal with another threat? Will the new reality of living through a pandemic adversely affect families with high exposure to the threat of armed conflict more (or perhaps less) than families with low exposure?

The COVID-19 outbreak began in Israel at the end of February 2020; on March 17, 2020, a general lockdown was declared and all schools and kindergartens were closed, as were non-essential businesses (23). The lockdown lasted until May 2020. During this time, families were instructed to stay indoors as much as possible and to avoid visiting their relatives or friends. These instructions were sudden and led to significant changes in the daily activities of all Israeli families. Most of the families experienced major interruptions in their daily routines, especially because the parents were expected to continue working from home while also taking care of their children (24). In a state of continuous lockdown, preliminary evidence showed that although young children were less vulnerable to the disease itself (i.e., showed fewer health problems caused by COVID-19) (25, 26), the pandemic seemed to have a major effect on their psychological wellbeing and negatively affected their socioemotional development and possibly their future developmental paths (26). More specifically, research indicated that children under the age of 6 years exhibited elevated levels of clinginess, distraction, and irritability during and after the lockdown (25). This may have been due to the unexpected shutdown of daycares and kindergartens with no clear prospect of returning as well as the prolonged stay-at-home social isolation with the inability to play outdoors (27, 28).

In addition, parents were also vulnerable and exhibited more psychological symptoms, such as anxiety and depression (29, 30). Furthermore, parents reported having elevated levels of stress and burnout during the lockdown, especially when parenting very young children (24). This may be explained by a major burden on parents due to the long hours they had to care for their children, with very limited support or assistance (26, 27, 31). Furthermore, many parents experienced additional stressors at a more general level, including the loss of their jobs or their need to alternate between work and homeschooling, as well as health concerns due to COVID-19 (28, 29, 32). Altogether, these cumulative stressors may have spilled over and also affected child wellbeing (31). More specifically, researchers have suggested that parental stress may play a significant role in child adjustment difficulties, meaning that children may be directly at risk not only due do the effects of the pandemic, but also indirectly through their parents' experience (27, 29, 31). Because parents may be preoccupied with other stressors caused by the pandemic, they may be less emotionally available to their young children (26, 31), thus being less capable to meet their children's needs and negatively affecting their wellbeing.

Families experiencing prolonged exposure to armed conflict may be at a double risk of being negatively affected by the external man-made stressor (i.e., missile attacks) and the natural disaster (i.e., COVID-19), both causing large-scale disruptions threatening their lives (26). Both parents and children are dealing with extreme changes in their daily lives as well as with mental stresses and fears. For families living in an armed-conflict area, the addition of the pandemic's new external stress may put further strain on the family system because the families may have fewer initial resources, increasing the risk of dysfunction (26). Thus, our first hypothesis proposed that for families living with high exposure to armed conflict, the adverse impact of the COVID-19 pandemic on family routine and relationships as well as child's behaviors would be stronger, compared to families living with low exposure.

It is important to consider individual differences in parent and child adjustment to stressors. For example, at times of extreme situations (such as armed conflict or a global pandemic), some people are negatively affected by these situations, whereas others show resilience and strength and even benefit from the unique circumstances (20, 26, 29). For example, being at home for a long time may strengthen the family bond, enable more parental support for children, and create more opportunities for parent-child interactions (29).

Therefore, the second goal of this study is to track individual differences and uncover possible risk factors that may be related to a family's adjustment to two stressors (exposure to armed conflict and COVID-19) that may elicit family dysfunction. Because parents and children have a mutual influence on family climate (13) and may differently experience the various stressors, individual differences in parental and child characteristics–in particular parental posttraumatic stress symptoms (PTSS) and child self-regulation–will be examined. The difference between PTSD and PTSS should be noted, with PTSD referring to a more clinical diagnosis of post-traumatic stress disorder, whereas an examination of PTSS makes it possible to broaden the observation in such a way that it emphasizes not only those who are clinically diagnosed with PTSD but also those experiencing post-traumatic stress symptoms. In this study, we chose to address participants' self-reports of post-trauma symptoms because they are merely a normative, non-clinical population, yet a population that suffers from prolonged exposure to armed conflict.

Recurrent findings in the area of exposure to armed conflict highlight the impairment of parental mental state and especially high levels of PTSS (16, 33). Findings also show that parental mental state was found to be associated with increased regulation difficulties among young children in armed-conflict areas, and this relation is mediated by maternal self-regulation tendencies (34). Consequently, a mother's PTSS may interfere with her capability to pause and approach her child in an adapted and attuned manner. For example, a mother exhibiting high levels of hyperarousal may have a decreased capacity to comfort her child while stressful or fearful situations occur (5). Moreover, traumatized parents who are overly preoccupied with their trauma and trauma-related issues may exhibit more difficulties in being present and emotionally available for their children, as well as difficulties in tolerating intense parent-child interactions (15. 20). In times of uncertainty (such as during the COVID-19 pandemic), the new reality may trigger a mother's PTSS, thus affect her parenting behaviors and thereby adversely affect her child's wellbeing and the entire family system.

Nevertheless, children are not passive participants in their families, but are active participants who respond in mutual interactions to the way parents regulate themselves (13, 35). Thus, children with dysregulation may negatively affect the parent (35). As previous studies show, children in armed-conflict areas exhibit more self-regulation difficulties, which are especially reflected in higher behavioral problems (3, 7). Therefore, we suggest that high levels of maternal PTSS and low levels of child self-regulation measured before the outbreak will act as risk factors for adverse effects of COVID-19 on families, and this effect will be more robust for families living in the high-exposure areas because they experience cumulative stress.

**Figure 1:**
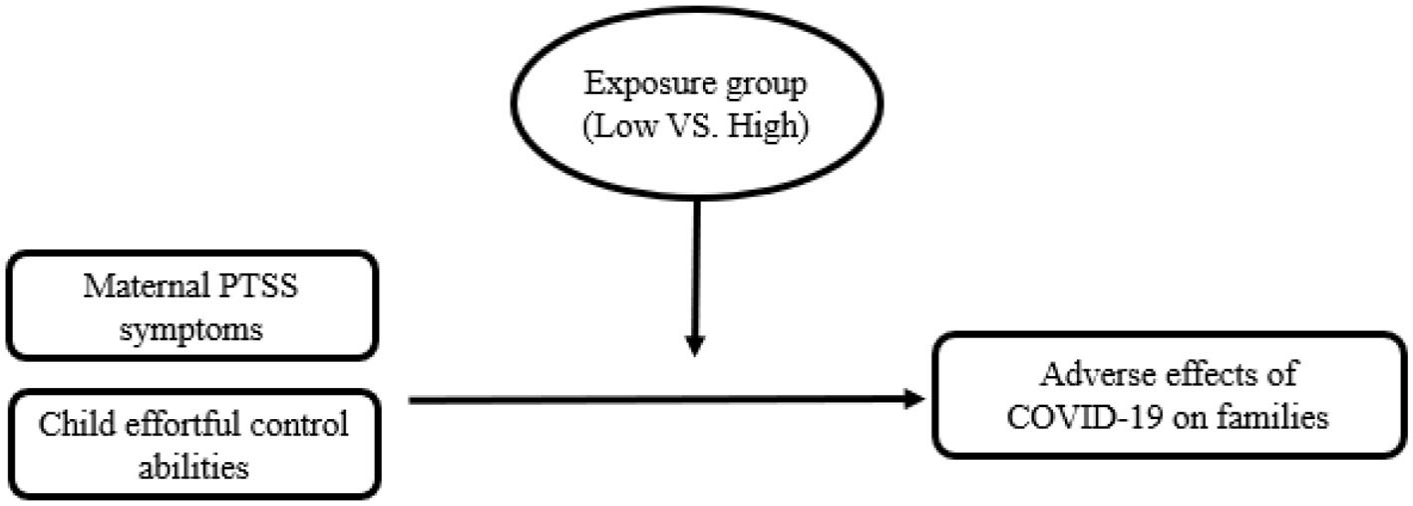
Moderation model for exposure group on the relation between maternal PTSS, child effortful control, and adverse effects of COVID-19 on families.

In the study, we emphasized two main ideas: the first is that cumulative stress caused by two external stressors–one man-made (exposure to armed conflict) and the other nature-made (COVID-19)–will be more robust than exposure to only one stressor (COVID-19). Thus, families with high exposure to an armed-conflict area will be more affected by COVID-19 outcomes, compared to families living in low-exposure areas. Second, we suggest that individual differences in risk factors may elicit a different yet still negative impact of COVID-19 on the family. Therefore, we propose that in the high-exposure group, higher levels of maternal PTSS and lower levels of child self-regulation tendencies, measured by child effortful control, will predict the adverse effects of COVID-19 lockdown on families (see Figure 1), such as changes in parenting or child's behaviors, parent-child relationship, parental mental health, or household rules Specifically, we hypothesized that:

1. There would be found group differences in the adverse effects of COVID-19 on families living in the two locations. Specifically, mothers in the high-exposure group would report higher levels of adverse effects of COVID-19 on the family, compared to mothers from the low-exposure group.
2. Increased maternal PTSS and lower levels of child effortful control (as an indicator of self-regulation) assessed prior to the pandemic will predict more adverse effects of COVID-19.
3. The links between both maternal PTSS and child self-regulation to adverse effects of COVID-19 will be moderated by exposure group. Specifically, in families living in the high-exposure area, mothers who have higher levels of PTSS and/or have children exhibiting lower levels of self-regulation tendencies prior to the outbreak of COVID-19 will experience more adverse effects of COVID-19, compared to parents and children in the low-exposure group.

## Materials and Methods

### Participants

Participants in this study included 118 mothers and their firstborn children aged 10–45 months (*SD* = 6.99) at Time 1 (T1) and 13–59 months of age (*SD* = 7.97) at Time 2 (T2); 53.4% were male. All families participated in the “Three to Four Study” examining familial changes upon the arrival of the second born. Data were included from two time points: (1) pre-COVID-19, and (2) toward the end of the lockdown of the first wave of the pandemic (May 2020). Inclusion criteria included intact families expecting their second child, singleton pregnancies, typically developed firstborns, and parents who were fluent in Hebrew. The sample included 40 mothers living in the Gaza vicinity (high-exposure group), and 78 mothers living in other areas of southern Israel (low-exposure groups). The exposure groups were defined by their distance from the Gaza Strip, with the high-exposure group including families living in localities within a range of up to 10 kilometers from the Gaza Strip and who had experienced armed conflict in this area for more than two decades. The low-exposure group included families that lived in other areas in the southern district of Israel. The high-exposure group was under constant exposure to missile attacks and other improvised explosive devices (attached to kites or balloons) that were launched from the Gaza Strip and that landed in residential areas, as well as daily military activity on the perimeter fence between the Gaza Strip and Israel. During the data collection period (October 2018 through May 2020), thousands of rockets were launched into the Gaza vicinity, and such incursions had become more frequent and unpredictable, with some periods characterized by hundreds of rockets per day launched from the Gaza Strip (21).

Demographic information concerning children's gender, children's age, mothers' age and mothers' education was reported, showing that 53.4% of the children were male, age range was 10–45 months (*SD* = 6.99) at Time 1 (T1) and 13–59 months of age (*SD* = 7.97) at Time 2 (T2). Mothers' mean age was 29.5 years (*SD* = 4.09), and most had higher levels of education (81.4%). Group differences in demographic variables were found only for child's age, such that children in the high-exposure group were younger than children in the low-exposure group, *t*_(116)_ = −2.47, *p* < 0.05, *M* = 22.9 months, *SD* = 6.75; *M* = 25.69, *SD* = 6.99; for high-exposure and low-exposure accordingly (see Table 1). Thus, age was controlled in all analyses. The examination of the associations between the demographic and the study variables revealed significant correlations between both child's age and mother's age with the COVID-19 adverse effect on families, such that the older the child or mother, the more adverse effects that were reported *(r* = 0.25, *p* <0.01; *r* = 0.23, *p* <0.05, accordingly).

**Table 1:** Group differences in study variables.

**Variable** **Mean (SD)**	**High-exposure group (*n* = 40)**	**Low-exposure group (*n* = 78)**	***t***
1. PTSS re-experience	1.79 (0.82)	1.40 (0.55)	2.66^**^
2. PTSS avoidance	1.66 (0.72)	1.34 (0.40)	2.64^*^
3. PTSS hyperarousal	1.76 (0.75)	1.74 (0.91)	0.11
4. Child effortful control	3.24 (0.69)	3.25 (0.64)	−0.08
5. Adverse effects of COVID-19	3.54 (0.77)	3.88 (0.71)	−2.4^*^
6. Child's age	22.10 (6.87)	25.38 (6.82)	−2.47^*^

* *p < 0.05*,

** *p < 0.01*.

### Procedure

The study was approved by the Clalit Health Services' Helsinki Ethics Committee and Ben-Gurion University of the Negev's Human Subjects Research Committee. Mothers were recruited through women's health centers, day care centers, and online advertisements. Home visits were conducted with interested families. At T1 mothers completed questionnaires (other maternal and child measures were taken but are not within the scope of this study). At T2, mothers were contacted *via* phone and asked to answer a survey concerning the adverse effects of COVID-19 on their family during the lockdown. Because the study is a longitudinal study that began before the outbreak of the COVID-19 pandemic, the time elapsed between T1 and T2 varied between families (*M* = 9.54 months, *SD* = 4.15 months).

### Materials

#### Maternal PTSS

The PTSD Checklist-Civilian version [PCL-C; (36)] was used to assess maternal PTSS related to exposure to armed conflict. This is a standardized self-report rating scale comprising 17 items that correspond to the key symptoms of PTSD, which are composite in three scales: re-experiencing (e.g., “Repeated, disturbing memories, thoughts, or images of a stressful experience from the past”; Cronbach alpha was high: α = 0.84), avoidance (e.g., “Avoiding *thinking about* or *talking about* a stressful experience from the past or avoiding having feelings related to it”;Cronbach alpha was high: α = 85) and hyperarousal (e.g., “Feeling jumpy or easily startled”; Cronbach alpha was mild: α = 0.64). Items were rated on how often the symptom affected the respondent in the past month, on a scale ranging from 1 (*not at all*) to 5 (*extremely*), providing a symptom-severity rating. The percentage of missing data for each scale was 5% or less, so the data were completed using the scale average. The questionnaire was validated for use in the Hebrew language [see (37, 38)].

#### Child Self-Regulation Tendencies

To assess child self-regulation, mothers completed the effortful control scale from the Early Childhood Behavior Questionnaire—very short form [ECBQ-VS; (39)]. This is a validated measure that consist of 12 items, rated on a 7-point scale from 1 (*never*) to 7 (*always*), concerning child effortful control tendencies (e.g., “When you were busy, how often did your child find another activity to do when asked?”; Cronbach alpha was mild: α = 0.68). The questionnaire was validated for use in the Hebrew language [see (40, 41)].

#### The Adverse Effects of COVID-19 on Families

Mothers were interviewed over the phone and were asked 8 questions (see Appendix) concerning the adverse effects of COVID-19 on their family during the lockdown, rated on a 7-point scale from 1 (*Much has changed for the better*) to 7 (*Much has changed for the worse*). The questionnaire consisted of questions regarding child, parent, and family changes in behaviors, relationships, and routines following the pandemic (e.g., “How much has the crisis affected your child's behavior?”; Cronbach alpha was moderate: α = 0.70). Items were averaged to create a single score. Higher scores reflected more adverse impact.

### Analytic Plan

First, preliminary analyses examining differences between high- and low-exposure groups as well as bivariate correlations were conducted. Next, an analysis of covariance (ANCOVA) test was conducted to test group differences for adverse effects of COVID-19 while controlling for child's age. Next, to test predictions of the independent variables on adverse effects, as well as the moderation effect by exposure group, the SPSS PROCESS macro (Model 1) was used, with a 95% bias-corrected bootstrapped confidence interval (CI) based on 2,000 bootstrap samples. Four separate models were tested, one for each predictor (i.e., re-experiencing, avoidance, hyperarousal, and child effortful control), predicting the adverse effects of COVID-19. Child's age was included as a covariate in all analyses. All continuous variables were first standardized. CIs that did not include zero indicated significant effects.

## Results

### Preliminary Analysis

To provide initial support for the model constructs, descriptive statistics and correlational analyses were first conducted (see Table 2). As seen, the three PTSS scales were significantly related to each other. In addition, a positive significant correlation was found between PTSS avoidance and adverse effects of COVID-19. Furthermore, mean-level differences between exposure groups for all the study's variables were examined using *t*-test analyses. Results revealed significant differences for PTSS re-experiencing, *t*_(116)_ = 2.66, *p* <0.05, and for PTSS avoidance, *t*_(116)_ = 2.64, *p* < 0.05, such that mothers from the high-exposure group reported having more re-experiencing symptoms (*M* = 1.79, *SD* = 0.82) and more avoidance symptoms (*M* = 1.66, *SD* = 0.72) compared to mothers from the low-exposure group (*M* = 1.40, *SD* = 0.55; *M* = 1.34, *SD* = 0.40, accordingly). However, no significant differences were found for PTSS hyperarousal, *t* (116) = 0.91, ns. Additionally, no significant differences were found for child effortful control tendencies, *t*_(116)_ = 0.94, ns.

**Table 2:** Means, standard deviations, and correlations for study variables.

**Variable**	**1**.	**2**.	**3**.	**4**.	**5**.	**Mean**	***SD***
1. PTSS re-experience	–					1.53	0.68
2. PTSS avoidance	0.45^**^	–				1.45	0.55
3. PTSS hyperarousal	0.34^**^	0.47^**^	–			1.75	0.86
4. Child effortful control	−0.11	−0.15	−0.02	–		3.24	0.66
5. Adverse effects of COVID-19	−0.004	0.20^*^	0.16	−0.17	–	4.04	0.80
6. Child's age	−0.16	−0.06	0.01	0.14	0.25^**^	24.27	6.99

* *p < 0.05*,

** *p < 0.01*.

### Main Analyses

To test the first hypothesis proposing group differences for adverse effects of COVID-19 on families between the exposure groups, an ANCOVA test was conducted while controlling for child's age. Contrary to expectations, no significant differences were found in maternal reports of the adverse effects of COVID-19 on families between the high- and low- exposure groups, *F*_(1, 115)_ = 1.04, ns.

To test our second and third hypotheses, proposing that maternal PTSS and child effortful control prior to the outbreak of COVID-19 would predict the adverse effects of COVID-19 on families, and that these predictions would be moderated by exposure group, multiple regression analyses were conducted. Results are presented in Table 3. Supporting the second hypothesis, results showed a significant effect for both avoidance symptoms, β = 1.08, *SE* = 0.39, *p* < 0.01, 95% CI (0.30, 1.86), and for hyperarousal symptoms, β = 0.77, *SE* = 0.34, *p* < 0.05, 95% CI (0.10, 1.44), meaning that for the entire sample, the more avoidance and hyperarousal symptoms mothers experienced prior to the outbreak of COVID-19, the more adverse effects of COVID-19 during the first wave of the pandemic were reported. Similarly, results for child effortful control indicated a significant effect, β = −1.06, *p* < 0.01, 95% CI (−1.79, −0.32), suggesting that lower scores in child effortful control prior to the outbreak predicted more adverse effects of COVID-19 on families during the first wave of the pandemic. However, no significant effect was found for PTSS re-experiencing, β = 0.20, *SE* = 0.34, ns, 95% CI (−0.49,0.89), meaning that re-experiencing symptoms did not predict the adverse effects of COVID-19 on families.

**Table 3:** Regression coefficients for moderation models predicting adverse effects of COVID-19 on families.

	**Adverse effects of COVID-19** ***R**^**2**^* **= 0.09**		
**Variable**	**β**	***SE***	***t***
**MODEL 1**			
Child's age	0.03^**^	0.01	2.87
Group	0.19	0.16	1.17
PTSS re-experiencing	0.20	0.35	0.58
Group × re-experiencing	−0.08	0.22	−0.37
Δ *R^2^* = 0.00			
**MODEL 2**			
Child's age	0.03^**^	0.01	2.80
Group	0.29	0.15	1.89
PTSS avoidance	1.08^**^	0.39	2.75
Group x avoidance	−0.51^*^	0.27	−1.90
Δ *R^2^* = 0.03			
**MODEL 3**			
Child's age	0.03^**^	0.01	2.76
Group	0.16	0.15	1.10
PTSS hyperarousal	0.77^*^	0.34	2.30
Group x hyperarousal	−0.36^*^	0.19	−1.91
Δ *R^2^* = 0.03			
**MODEL 4**			
Child's age	0.03^**^	0.01	3.25
Group	0.14	0.15	0.95
Effortful control	−1.06^**^	0.37	−2.86
Group x effortful control	0.49^*^	0.22	2.80
Δ *R^2^* = 0.04			

* *p <0.05*,

** *p <0.01*.

*Model 1 = Maternal PTSS re-experiencing scale as a predictor; Model 2 = Maternal PTSS avoidance scale as a predictor; Model 3 = Maternal PTSS hyperarousal scale as a predictor; Model 4 = Child effortful control as a predictor*.

**Figure 2:**
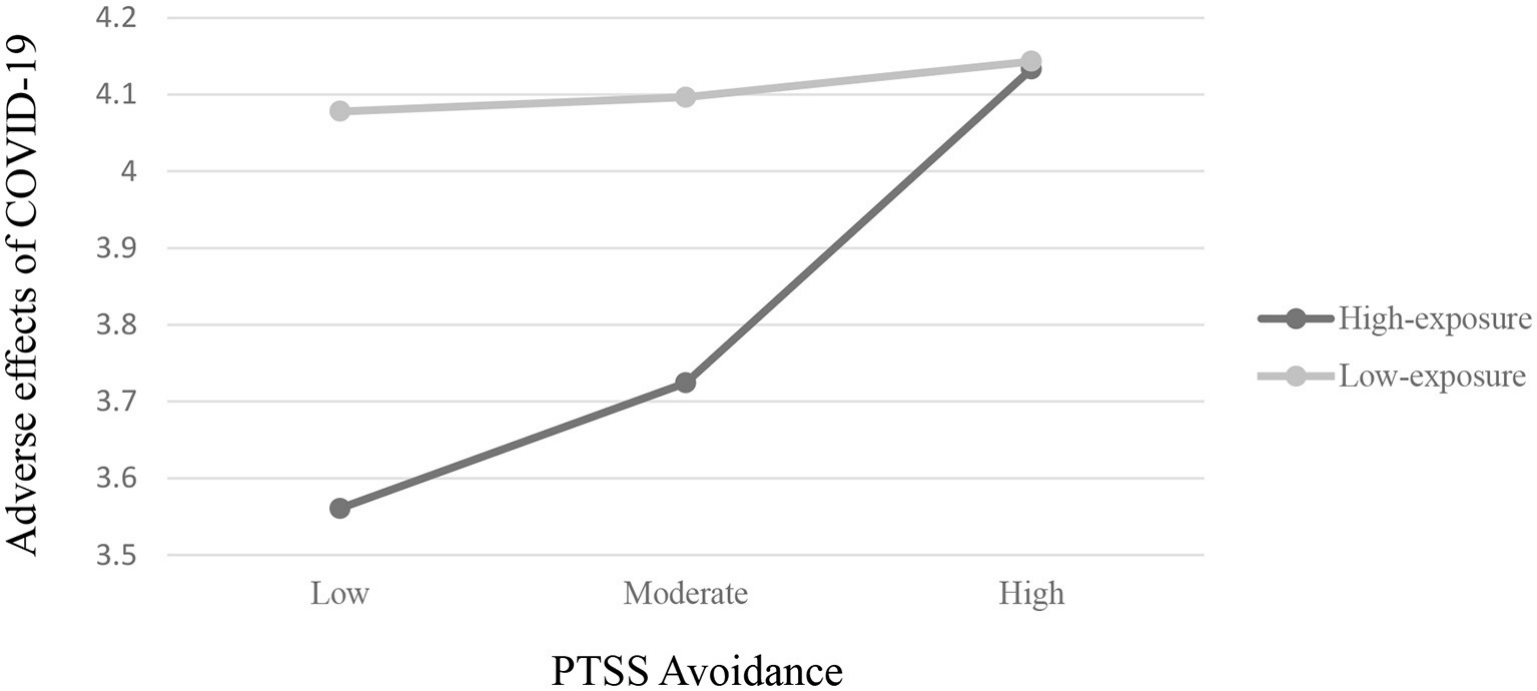
Two-way interaction of maternal PTSS avoidance and exposure group on adverse effects of COVID-19 on families.

**Figure 3:**
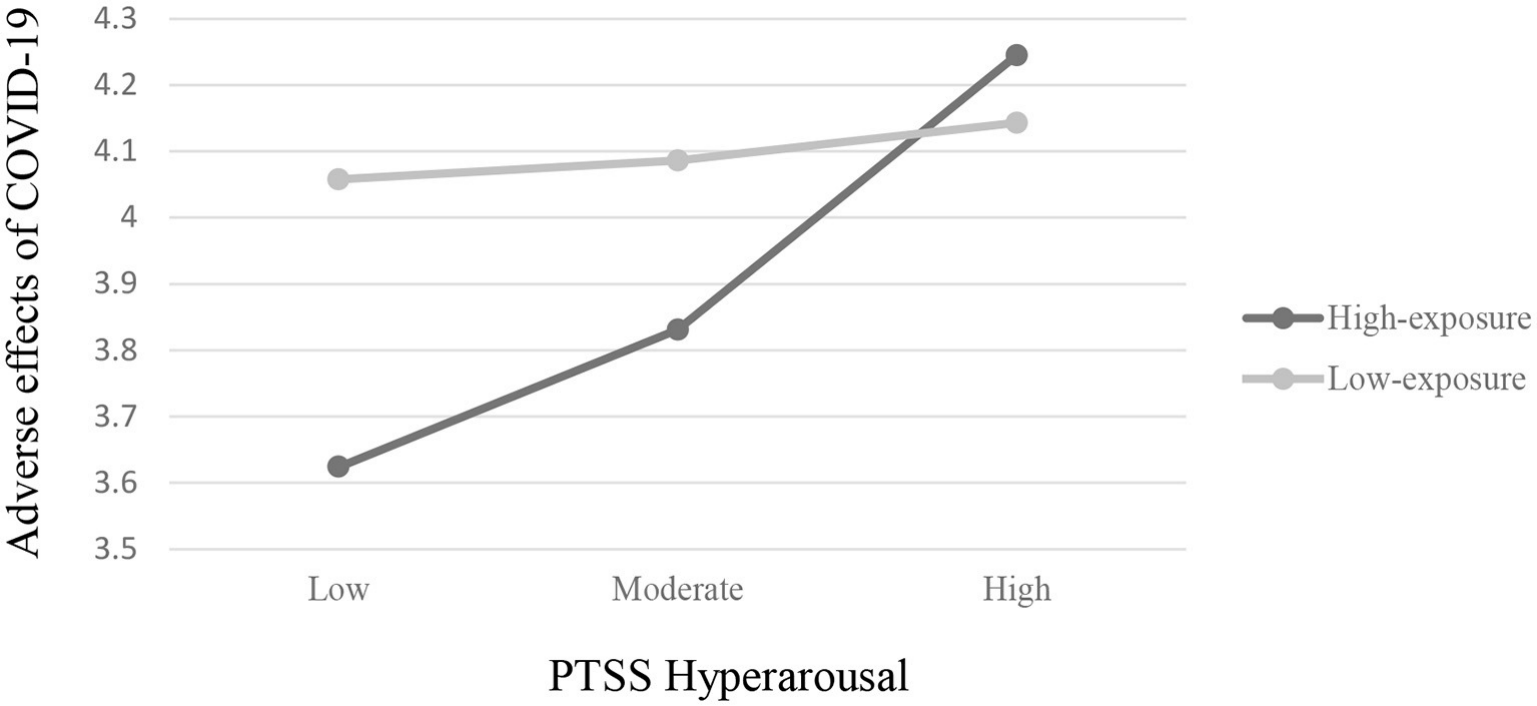
Two-way interaction of maternal PTSS hyperarousal and exposure group on adverse effects of COVID-19 on families.

Next, the third hypothesis proposing a moderation effect–such that exposure group would moderate the prediction of maternal PTSS as well as child effortful control tendencies on adverse effects of COVID-19–was partially supported. Interaction effects between maternal avoidance symptoms and group (high/low exposure) were significant (β = −0.51, *SE* = 0.27, *p* < 0.05, 95% CI [−1.04, 0.02]), as well as for hyperarousal symptoms and group (β = −0.36, *SE* = 0.19, *p* ≤ 0.06, 95% CI [−0.73,0.01]), meaning that group (high/low exposure) moderated the relation between both scales and the adverse effects of COVID-19. *Post-hoc* analyses revealed that for the high-exposure group, there was a significant effect for both maternal PTSS avoidant symptoms (β = 0.58, *SE* = 0.16, *p* < 0.001, 95% CI [0.25,0.90]) and maternal PTSS hyperarousal symptoms (β = 0.41, *SE* = 0.16, *p* < 0.05, 95% CI [0.09,0.73]) on the adverse effects of COVID-19, such that in families living in the high-exposure area, mothers who had less avoidant symptoms and/or less hyperarousal symptoms prior to the outbreak of COVID-19 experienced *less* adverse effects of COVID-19 during the first wave of the pandemic, than mothers from the high exposure group who were high in these scales. These links *were not found* among families living in low-exposure areas (β = 0.06, *SE* = 0.21, ns., 95% CI [−0.35,0.48]; β = 0.06, *SE* = 0.09, ns., 95% CI [−0.13,0.24] for maternal PTSS avoidant and hyperarousal symptoms, accordingly). Results are presented in Figures 2, 3. However, contrary to the hypothesis, no significant interaction effect was found between maternal re-experiencing symptoms and group (β = −0.08, *SE* = 0.22, ns, 95% CI [−0.52,0.36]), meaning that maternal re-experiencing symptoms predicted the adverse effects of COVID-19 in the same manner for the two exposure groups.

**Figure 4:**
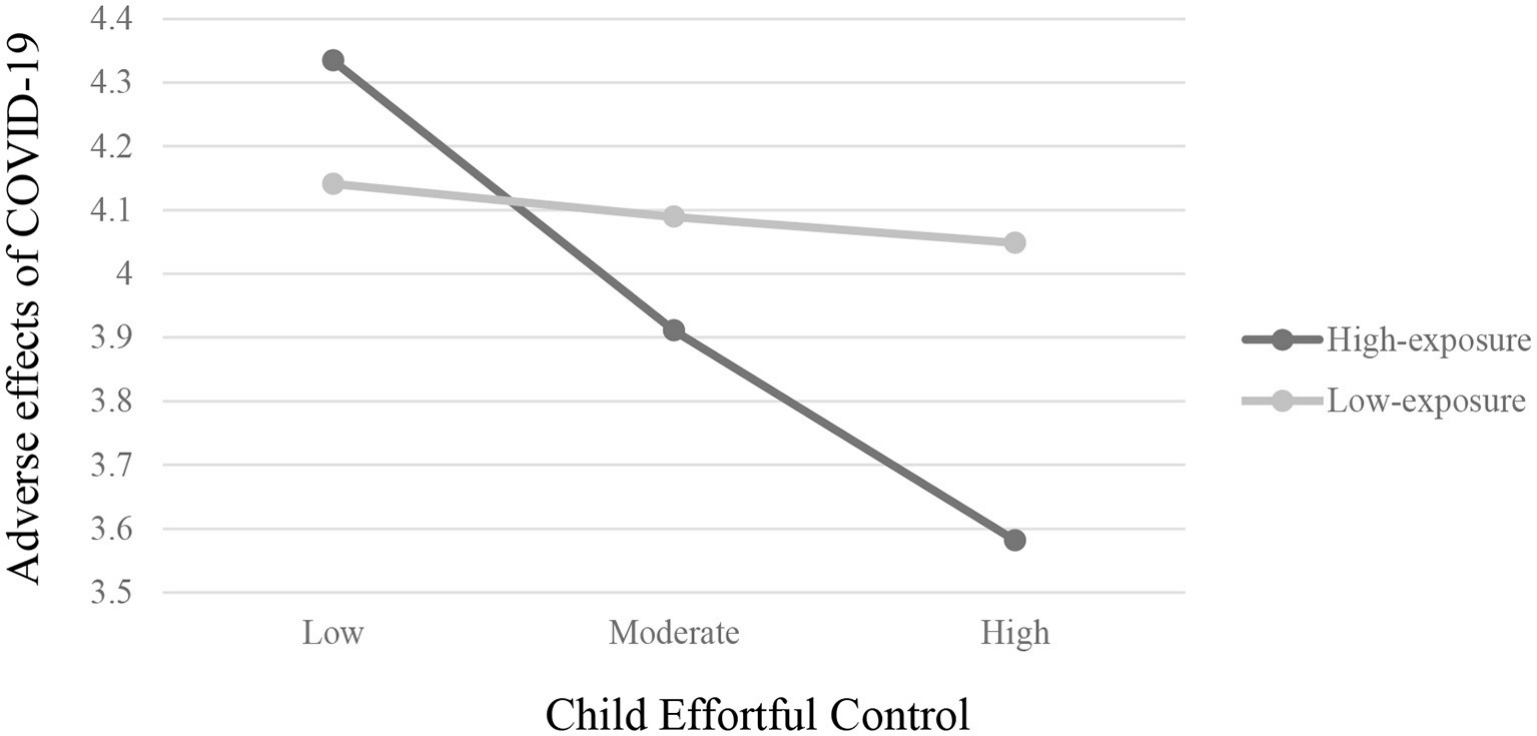
Two-way interaction of child effortful control and exposure group on adverse effects of COVID-19 on families.

Finally, a significant interaction effect was found between child effortful control tendencies and group (β = 0.49, *SE* = 0.22, *p* < 0.05, 95% CI [0.06,0.92]). *Post hoc* analyses revealed that among the high-exposure group, there was a significant effect for child effortful control (β = −0.56, *SE* = 0.17, *p* < 0.01, 95% CI [−0.91, −0.22]) on the adverse effects of COVID-19, such that in families living in the high-exposure area (whose children exhibited higher scores in effortful control prior to the outbreak of COVID-19) experienced the adverse effects of COVID-19 less during the first wave of the pandemic. This link was not found among families from the low-exposure group (β = −0.07, *SE* = 0.13, ns., 95% CI [−0.33,0.19]). Results are presented in Figure 4.

## Discussion

When faced with inevitable large-scale stressors, most families are affected, but variance may occur in the extent to which it affects families. The aim of this study was to examine the extent to which families already living in a high-risk area–exposed to uncertainty and chronic stress–are adversely affected by a new external threat (COVID-19), which adds more stress to the family setting. Moreover, the role of maternal PTSS and child self-regulation tendencies–assessed prior to the stressors as risk factors–were examined. Because this study is part of a research project that began before the outbreak of the COVID-19 pandemic, we had the unique opportunity to investigate these questions.

The first hypothesis proposing that families living in areas with high exposure to armed conflict would experience more adverse effects of COVID-19 compared to families living in low-exposure areas was not supported. No differences were found between the exposure groups in the adverse effects of COVID-19 variable. Several explanations may be proposed. First, it is possible that government guidelines led to a general reduction in stress (caused by the unpredictable exposure to missile attacks) among mothers from the high-exposure group because they were instructed to stay home. Thus, mothers knew that even if an unexpected attack occurred, they and their children would be close to a protected shelter. This is somewhat different from the regular routine of life in armed-conflict areas where parents and children may be exposed to an unpredicted missile attack while outdoors (e.g., at the park or driving home) and far from a protected shelter (42). Furthermore, interestingly, during the first lockdown (March-May 2020) almost no rockets were launched toward the Gaza vicinity (21). In fact, this was the first time in the past two years that families living in the Gaza vicinity experienced a quiet period of two months. Thus, an alternative explanation may be that the lack of differences between the groups stems from the fact that during this period the residents did not directly experience stress from missile attacks and therefore their stress level was lower in general as a group. This idea is supported by previous findings indicating that although some people may have established anxiety and distinct symptoms of PTSS, there are others for whom anxiety and PTSS persist only in times or areas where their chances of exposure are high (42). Conversely, research has found that when these people are physically in another area of the country where the threat is low, these symptoms disappear almost completely. Therefore, it may be that for parents in the high-exposure group, the abrupt decline in the level of danger reduced the overall stress levels and thus reduced the effect of exposure, which is reflected in the insignificant differences between the exposure groups and the adverse effects of the COVID-19 variable.

Additionally, hypothesis proposing that increased maternal PTSS and lower levels of child effortful control assessed prior to the pandemic would predict more adverse effects of COVID-19, particularly to mothers from the high-exposure group, was partially supported. Supporting our hypothesis, mothers from the high-exposure group having higher levels of maternal trauma symptoms and/or lower levels of child's effortful control showed more adverse effects, from COVID-19, than mothers having less trauma symptoms. Interestingly, mothers from the low exposure group also reported higher levels of adverse effect from COVID-19. In other words, the more striking finding was that families from the high exposure group whose mothers reported less trauma symptoms and/or had children with higher levels of effortful control, were significantly less affected from the COVID-19 lockdown consequences. These results proposes that this group may be perceived as a more “resilient group.”

Moreover, these findings strengthen the importance of an in-depth consideration of individual differences within the exposure groups. Thus, the examination of the differences between the groups did not reveal that the high-exposure group, *per se*, was related to more negative/positive COVID-19 effects, but rather, it was the combination of high-exposure and maternal PTSS or child executive functioning that buffer the risk for more adverse effects of COVID-19 on the family. It is possible that the adjustment to the life in stressful armed conflict zones, when having good mental health and regulated children, act as resilient factor that enable families to adjust to new stressful condition, such as the COVID-19 pandemic. As for families from the low exposure group, these findings go along with general finding from around the world, showing adverse effects of the first wave lockdown on family settings, as well as parental and child mental health (43).

Furthermore, several possible explanations may apply to the results indicating of high adverse effect for families living in high-exposure group whose mothers reported of more difficult mental state and had less regulate children. First, these findings are consistent with previous research indicating that families who carry cumulative and chronic stress are more vulnerable when confronting a new threat because they have fewer internal resources to handle cascading external threats, which spillover to family functioning (26, 32). Another possible explanation may be that in the context of maternal PTSS, when mothers struggle with functioning in the family setting, it may affect their capacity to buffer the negative effects on their children (caused by the pandemic) because they are themselves overwhelmed (26). Supporting this idea, previous studies have indicated that traumatized parents who are overly occupied with their own trauma may exhibit more difficulties with being present and emotionally available for their children, as well as in tolerating intense parent-child interactions (15, 20). In the context of the pandemic, mothers were required to spend long hours with their children and, if they already had PTSS, may have experienced the prolonged stay with their children as overwhelming and unbearable. In a state of pattern of avoidance or hyperarousal, their capacity to provide an appropriate emotional response to their children may be impaired because they may fail to regulate their own emotions (20). This idea should be taken with limited caution, as in the present study no association was found between maternal PTSS and mothers' report of their child's regulation difficulties. This may reflect either no objective relation between maternal PTSS and child's regulation difficulties, or a specific noise in the current data as these scales had low Cronbach's alphas. Finally, an additional explanation may be that, in the context of armed conflict, previous studies suggest that the link between maternal PTSS and child wellbeing is mediated by maternal self-regulation as well as by maternal parenting practices (15, 34). Thus, when a mother has PTSS, she may find it difficult to regulate her distress because her mental resources are limited, leading to parenting practices that are less emotionally available and more hostile (15). In the context of facing the new external threat of COVID-19, researchers have suggested that parents who experience cumulative stress are more likely to exhibit rigid and abusive parenting behaviors (30). Further research is needed to understand the role of parenting practices as mediators in the link between maternal mental state and family functioning during the COVID-19 pandemic.

Furthermore, our findings suggest that low child self-regulation tendencies predicted adverse effects of COVID-19, especially in families from the high-exposure group. These findings highlight the impact of children's characteristics on the family system, as proposed by the transactional model (35) and the family system models (44). Through mutual and reciprocal exchanges, children influence their parents and their families and are influenced by them. Moreover, previous studies suggest that young children have fewer personal resources to help adjust to the many changes the pandemic brings to their daily routine (31), thus there is a need for a parent who is emotionally available to the child and is capable to manage or contain his or her negative feelings toward the new situation. However, in the context of living in an armed-conflict area, parents may be less available, especially when the child exhibits low self-regulation, which may add more stress to the family system. Thus, it is likely that during a prolonged stay-at-home situation when a child exhibits less self-regulation, the entire family will be affected and thus the family will be more vulnerable to the adverse effects of COVID-19, especially in the context of armed conflict.

### Limitation and Future Directions

Several limitations to our research should be noted. First, this study is based on maternal reports because no in-person child assessments or observations were allowed due to COVID-19 social-distancing regulations. Future studies may use online observations and assessments that may allow for a more objective examination regarding family functioning, child self-regulation, and the parent-child relationship. Second, the sample in this study was relatively small, especially in the high-exposure group. Moreover, Cronbach's alphas for hyperarousal and effortful control scales were rather small. Thus, all moderation tests should be addressed with caution. Replicating this study with a larger sample may uncover possible mechanisms of maternal self-regulation and shed light on optional interventions that can be derived from the findings. Finally, maternal PTSS was measured using a self-report questionnaire. Future studies should use a clinical interview of maternal PTSS because it may provide a more objective assessment and enable a deeper and broader understanding of parenting under cumulative stress, resulting in a stronger validity to the research findings.

## Conclusions

Studies concerning the adverse effect of lockdown on families with very young children are scarce. The results of this study should encourage further research that will specifically examine the adverse effects of the COVID-19 pandemic on early childhood as well as investigate the impact of the prolonged lockdown on family functioning and parental mental state and the way that these consequences cascade on future developmental pathways.

To date, several studies that have investigated the impact of the pandemic on family functioning and parental and child wellbeing conclude that a beneficial home climate is critical for a child's capacity to cope during the pandemic. Therefore, a supportive environment that adapts itself to the child's needs may serve as a protective factor against the psychological effects of the lockdown (28). However, parents who have been experiencing other stressors, such as continuous exposure to armed conflict, may have limited resources due to a depletion resulting from prolonged exposure to stress. Therefore, additional external stress brought on by the COVID-19 pandemic may have a more adverse effect. Yet, there are individual differences and unique characteristics that may facilitate, or alternatively, put the family at further risk when faced with a stressor. In our study, we showed that maternal PTSS (particularly avoidant and hyperarousal symptoms) and low child self-regulation tendencies acted as risk factors, putting mothers and children at higher risk of experiencing more adverse effects with COVID-19. Thus, clinician and community aid services working with families who live in armed-conflict areas should focus on the times when families experience additional stressors and especially focus on providing special care to families with mothers experiencing PTSS or children exhibiting self-regulation difficulties. These families need specific and systemic support to continue functioning in an optimal manner when experiencing additional stressors, such as the COVID-19 pandemic.

## Data Availability Statement

The raw data supporting the conclusions of this article will be made available by the authors, without undue reservation.

## Ethics Statement

The study was approved by the Clalit Helsinki Review Board and Ben-Gurion University of the Negev's Human Subjects Research Committee. Written informed consent to participate in this study was provided by the participants' legal guardian/next of kin.

## Author Contributions

PY, AP, KD-D, and NA-P contributed to conception and design of the study. AH, GB, and MF helped recruiting the participants in their clinics. All authors contributed to manuscript revision, read, and approved the submitted version.

## Funding

This research was funded by a grant from the U.S.-Israel Binational Science Foundation (Grant 2016023).

## Conflict of Interest

The authors declare that the research was conducted in the absence of any commercial or financial relationships that could be construed as a potential conflict of interest.

## Publisher's Note

All claims expressed in this article are solely those of the authors and do not necessarily represent those of their affiliated organizations, or those of the publisher, the editors and the reviewers. Any product that may be evaluated in this article, or claim that may be made by its manufacturer, is not guaranteed or endorsed by the publisher.

## Appendix

### Adverse Effects of COVID-19 Questions

1. To what extent did the COVID-19 crisis affect your child's behavior?
2. To what extent did the COVID-19 crisis affect your financial situation?
3. To what extent did the COVID-19 crisis affect your work?
4. To what extent did the COVID-19 crisis affect your household behavior (home rules)?
5. To what extent did the COVID-19 crisis affect your emotional state?
6. To what extent did the COVID-19 crisis affect your parenting behaviors and feelings of efficiency?
7. To what extent did the COVID-19 crisis affect your mother-child relationship?
8. To what extent did the COVID-19 crisis affect your relationship with grandparents?

## References

[1] Kadir A Shenoda S Goldhagen J. Effects of armed conflict on child health and development: a systematic review. PLoS ONE. (2019) 14:1–37. 10.1371/journal.pone.0210071 30650095 PMC6334973

[2] Cohen E Shulman C. Mothers and toddlers exposed to political violence: severity of exposure, emotional availability, parenting stress, and toddlers' behavior problems. J Child Adolesc Trauma. (2019) 12:131–40. 10.1007/s40653-017-0197-1 32318186 PMC7163821

[3] Pat-Horenczyk R Schiff M. Continuous traumatic stress and the life cycle: exposure to repeated political violence in Israel. Curr Psychiatr Reps. (2019) 21:8. 10.1007/s11920-019-1060-x 31264027

[4] Keresteš G. Children's aggressive and prosocial behavior in relation to war exposure: Testing the role of perceived parenting and child's gender. Int J Behav Develop. (2006) 30:227–39. 10.1177/0165025406066756

[5] Pat-Horenczyk R Ziv Y Asulin-Peretz L Achituv M Cohen S Brom D. Relational trauma in times of political violence: continuous versus past traumatic stress. Peace Conflict: J Peace Psychol. (2013) 19:125–37. 10.1037/a0032488

[6] Lahad M Leykin D. Ongoing exposure versus intense periodic exposure to military conflict and terror attacks in Israel. J Traumatic Stress. (2010) 23:691–8. 10.1002/jts.20583 21171129

[7] Slone M Mann S. Effects of war, terrorism and armed conflict on young children: A systematic review. Child Psychiatr Hum Develop. (2016) 47:950–65. 10.1007/s10578-016-0626-7 26781095

[8] Masten AS Narayan AJ. Child development in the context of disaster, war, and terrorism: pathways of risk and resilience. Ann Rev Psychol. (2012) 63:227–57. 10.1146/annurev-psych-120710-100356 21943168 PMC5858878

[9] Sadeh A Hen-Gal S Tikotzky L. Young children's reactions to war-related stress: a survey and assessment of an innovative intervention. Pediatrics. (2008) 121:46–53. 10.1542/peds.2007-1348 18166556

[10] Chu AT Lieberman AF. Clinical Implications of Traumatic Stress from Birth to Age Five. Ann Rev Clinic Psychol. (2010) 6:469–94. 10.1146/annurev.clinpsy.121208.131204 20192799

[11] Lieberman AF. Infants remember: war exposure, trauma, and attachment in young children and their mothers. J Am Acad Child Adolesc Psychiatr. (2011) 50:640–1. 10.1016/j.jaac.2011.04.009 21703490

[12] Cummings EM Merrilees CE Taylor LK Mondi CF. Developmental and social–ecological perspectives on children, political violence, and armed conflict. Develop Psychopathol. (2017) 29:1–10. 10.1017/S0954579416001061 27869066

[13] Halevi G Djalovski A Kanat-Maymon Y Yirmiya K Zagoory-Sharon O Koren L. The social transmission of risk: Maternal stress physiology, synchronous parenting, and well being mediate the effects of war exposure on child psychopathology. J Abnorm Psychol. (2017) 126:1087–103. 10.1037/abn0000307 29154569

[14] Betancourt TS Khan KT. The mental health of children affected by armed conflict: Protective processes and pathways to resilience. Int Rev Psychiatr. (2008) 20:317–28. 10.1080/09540260802090363 18569183 PMC2613765

[15] Zamir O Gewirtz AH Dekel R Lavi T Tangir G. Mothering under political violence: post-traumatic symptoms, observed maternal parenting practices and child externalising behaviour. Int J Psychol. (2020) 55:123–32. 10.1002/ijop.12557 30537100

[16] Devakumar D Birch M Osrin D Sondorp E Wells JCK. The intergenerational effects of war on the health of children. BMC Med. (2014) 12:1–15. 10.1186/1741-7015-12-57 24694212 PMC3997818

[17] Feldman R Vengrober A Eidelman-Rothman M Zagoory-Sharon O. Stress reactivity in war-exposed young children with and without posttraumatic stress disorder: relations to maternal stress hormones, parenting, and child emotionality and regulation. Develop Psychopathol. (2013) 25:943–55. 10.1017/S0954579413000291 24229541

[18] Pat-Horenczyk R Achituv M Kagan Rubenstein A Khodabakhsh A Brom D Chemtob C. Growing up under fire: building resilience in young children and parents exposed to ongoing missile attacks. J Child Adolesc Trauma. (2012) 5:303–14. 10.1080/19361521.2012.719595

[19] Sagi-Schwartz A. Children of war and peace: a human development perspective. J Conflict Resol. (2012) 56:933–51. 10.1177/00220027124461289170238

[20] Eltanamly H Leijten P Jak S Overbeek G. Parenting in times of war: a meta-analysis and qualitative synthesis of war exposure, parenting, and child adjustment. Trauma Viol Abuse. (2019) 19:1–14. 10.1177/1524838019833001 30852950 PMC7675766

[21] Israeli Home Front Command. Alert History. (2020). Available online at: https://info.oref.org.il/12481-he/Pakar.aspx

[22] Feldman R Vengrober A. Posttraumatic stress disorder in infants and young children exposed to war-related trauma. J Am Acad Child Adolesc Psychiatr. (2011) 50:645–58. 10.1016/j.jaac.2011.03.001 21703492

[23] Birenbaum-Carmeli D Chassida J. Covid-19 in Israel: Socio-demographic characteristics of first wave morbidity in Jewish and Arab communities. Int J Equity Health. (2020) 19:1–13. 10.1186/s12939-020-01269-2 32907584 PMC7480661

[24] Griffith AK. Parental burnout and child maltreatment during the COVID-19 pandemic. J Fam Violence. (2020) 2:1–7. 10.1007/s10896-020-00172-2 32836736 PMC7311181

[25] Jiao WY Wang LN Liu J Fang SF Jiao FY Pettoello-Mantovani M. Behavioral and emotional disorders in children during the COVID-19 epidemic. J Pediatrics. (2020) 221:264–6. 10.1016/j.jpeds.2020.03.013 32248989 PMC7127630

[26] Masten AS Motti-Stefanidi F. Multisystem resilience for children and youth in disaster: Reflections in the context of COVID-19. Adversity Resil Sci. (2020) 20:95–106. 10.1007/s42844-020-00010-w 32838305 PMC7314620

[27] Morelli M Cattelino E Baiocco R Trumello C Babore A Candelori C. Parents and children during the COVID-19 lockdown: the influence of parenting distress and parenting self-efficacy on children's emotional well being. Front Psychol. (2020) 11:1–10. 10.3389/fpsyg.2020.584645 33123063 PMC7574609

[28] Wang G Zhang Y Zhao J Zhang J Jiang F. Mitigate the effects of home confinement on children during the COVID-19 outbreak. The Lancet. (2020) 395:945–7. 10.1016/S0140-6736(20)30547-X 32145186 PMC7124694

[29] Achterberg M Dobbelaar S Boer O Crone EA. Home lockdown : Bloom or Boom? Perceived stress as mediator for longitudinal effects of the COVID-19 lockdown on wellbeing of parents and children. PsyArXiv. (2020) 20:3. 10.31234/osf.io/pj3sg PMC7859207 33536464

[30] Brown SM Doom JR Lechuga-Peña S Watamura SE Koppels T. Stress and parenting during the global COVID-19 pandemic. Child Abuse Neglect. (2020) 6:104699. 10.1016/j.chiabu.2020.104699 32859394 PMC7440155

[31] Spinelli M Lionetti F Pastore M Fasolo M. Parents' stress and children's psychological problems in families facing the COVID-19 outbreak in Italy. Front Psychol. (2020) 11:1–7. 10.3389/fpsyg.2020.01713 32719646 PMC7350926

[32] Brock RL Laifer LM. Family science in the context of the COVID-19 pandemic: solutions and new directions. Family Process. (2020) 59:1007–17. 10.1111/famp.12582 32663330 PMC7405324

[33] Goral A Lahad M Aharonson-Daniel L. Differences in posttraumatic stress characteristics by duration of exposure to trauma. Psychiatry Res. (2017) 258:101–7. 10.1016/j.psychres.2017.09.079 28992546

[34] Pat-Horenczyk R Cohen S Ziv Y Achituv M Asulin-Peretz L Blanchard TR. Emotion regulation in mothers and young children faced with trauma. Infant Mental Health J. (2015) 36:337–348. 10.1002/imhj.21515 25941026

[35] Sameroff A. A unified theory of development: a dialectic integration of nature and nurture. Child Develop. (2010) 81:6–22. 10.1111/j.1467-8624.2009.01378.x 20331651

[36] Weathers FW Litz BT Herman D Huska J Keane T. The PTSD Checklist for DSM-5 (PCL-5). The PTSD Checklist—Civilian Version (PCL–C)., The PTSD Checklist—Civilian Version (PCL–C). Bosto (1994).

[37] Besser A Zeigler-Hill V Weinberg M Pincus AL Neria Y. Intrapersonal resilience moderates the association between exposure-severity and ptsd symptoms among civilians exposed to the 2014 Israel–Gaza conflict. Self Identity. (2015) 14:1–15. 10.1080/15298868.2014.966143

[38] Besser A Neria Y. PTSD symptoms, satisfaction with life, and prejudicial attitudes toward the adversary among Israeli civilians exposed to ongoing missile attacks. J Traumatic Stress. (2009) 22:268–75. 10.1002/jts.20420 19593806

[39] Putnam SP Gartstein MA Rothbart MK. Measurement of fine-grained aspects of toddler temperament : the early childhood behavior. Questionnaire. (2006) 29:386–401. 10.1016/j.infbeh.2006.01.004 17138293 PMC4334385

[40] Abramson L Paz Y Knafo-Noam A. From negative reactivity to empathic responding: Infants high in negative reactivity express more empathy later in development, with the help of regulation. Developmental Sci. (2019) 22:1–17. 10.1111/desc.12766 30339317

[41] Harel-Gadassi A Friedlander E Yaari M Bar-Oz B Eventov-Friedman S Mankuta D. Do developmental and temperamental characteristics mediate the association between preterm birth and the quality of mother-child interaction? Infant Behav Dev. (2020) 58:101421. 10.1016/j.infbeh.2020.101421 32135402

[42] Diamond GM Lipsitz JD Fajerman Z Rozenblat O. Ongoing traumatic stress response (OTSR) in Sderot, Israel. Professional Psychol Res Pract. (2010) 41:19–25. 10.1037/a0017098

[43] Verger NB Urbanowicz A Shankland R McAloney-Kocaman K. Coping in isolation: Predictors of individual and household risks and resilience against the COVID-19 pandemic. Soc Sci Human Open. (2021) 20:100123. 10.1016/j.ssaho.2021.100123

[44] Minuchin P. Families and individual development: Provocations from the field of family therapy. Child Develop. (1985) 56:289–302. 10.2307/1129720 3886321

